# Outcomes indicators and a risk classification system for spinal manipulation under anesthesia: a narrative review and proposal

**DOI:** 10.1186/s12998-018-0177-z

**Published:** 2018-03-08

**Authors:** Dennis DiGiorgi, John L. Cerf, Daniel S. Bowerman

**Affiliations:** 1 Consultant Practice- Whitestone, NY, USA; 2 Clinical and Consultant Practice- Jersey City, NJ, USA; 3 Clinical and Consultant Practice- Philadelphia, PA, USA

**Keywords:** Manipulation under anesthesia, Spine, Medical evidence, Risk, Contraindications, Precautions, Outcomes, Informed consent

## Abstract

Over a period of decades chiropractors have utilized spinal manipulation under anesthesia (SMUA) to treat chronic back and neck pain. As an advanced form of manual therapy, SMUA is reserved for the patient whose condition has proven refractory to office-based manipulation and other modes of conservative care. Historically, the protocols and guidelines put forth by chiropractic MUA proponents have served as the clinical compass for directing MUA practice. With many authors and MUA advocates having focused primarily on anticipated benefit, the published literature contains no resource dedicated to treatment precautions and contraindications. Also absent from current relevant literature is acknowledgement or guidance on the preliminary evidence that may predict poor clinical outcomes with SMUA. This review considers risk and unfavorable outcomes indicators in therapeutic decision making for spinal manipulation under anesthesia. A new risk classification system is proposed that identifies patient safety and quality of care interests for a procedure that remains without higher-level research evidence. A scale which categorizes risk and outcome potential for SMUA is offered for the chiropractic clinician, which aims to elevate the standard of care and improve patient selection through the incorporation of specific indices from existing medical literature.

## Background

Since the 1930’s, many forms of facilitated manipulation of the spine have been reported in the literature, with various anesthetic/sedative agents and techniques used [1]. In the 1990’s, in managing patients with chronic spine pain and related dysfunction, chiropractors began utilizing the conscious sedation variety of manipulation under anesthesia (MUA) [1]. During that era, the standards and protocols of the National Academy of MUA Physicians (NAMUAP) [2] were popularized and relied upon by many in determining clinical eligibility for MUA. A revised version of the original NAMUAP standards and protocols was put forth in 2012, as adopted by the newly established American Association of Manipulation Under Anesthesia Providers (AAMUAP). That document subsequently underwent a consensus-based review, in developing recommendations for the contemporary practice of MUA [3].

What may render a clinical guideline useful for individual patients is its reliance upon valid evidence in establishing decision points and risks of care [4]. An appraisal of risk is essential to clinical guideline development [5–7]. Estimations of the balance of benefits against risks or harms helps to ensure guideline credibility for stakeholders [8]. A significant limitation with current SMUA guidelines, and the collective scientific knowledge on this subject, is inadequate recognition of the risk profiles and outcomes indicators that contribute to patient selection. This limitation relates to incomplete development of the relative/absolute contraindications to care and several known clinical, diagnostic, and litigation-related factors which may weigh against the option of SMUA.

For spinal manipulation under anesthesia, what remains ambiguous despite existing guidelines is when to move a patient toward treatment. The heterogeneity of primary research and the presence of mostly lower-level evidence pose inherent challenges to clinical decision making [9]. Moreover, the current SMUA literature and association-based protocol documents are without a corresponding evidence-informed resource that elucidates risk versus benefit. Even when individual patients have undergone all appropriate studies [10], weak levels of evidence for SMUA can lead to uncertainty in selection, dosing and patient safety. Although rare, sentinel events have been reported [11–13]. It is with a rise in SMUA utilization in the United States in recent years that a constructive and critical analysis of these matters becomes essential. This paper reviews the evidence for SMUA in establishing a scale for the chiropractic clinician which categorizes risk and outcome potential. It considers risk and unfavorable outcomes indicators in the development of a risk classification system that identifies patient safety and quality of care interests for this advanced form of treatment. As such, it calls for an elevation in the standard of care and improved patient selection through the incorporation of specific indices from existing medical literature.

## The principle of spinal adhesions

Within the existing SMUA literature, the principal mechanism theorized for procedural effectiveness is the disruption of soft tissue adhesions about the axial spine [2, 14–17]. In 1948, Clybourne proposed that the cases most amenable to MUA treatment were those involving joint adhesions, with limited movement in all directions [14]. He posited that the careful selection of cases was the product of an in-depth knowledge of joint anatomy and pathology [14]. However, he acknowledged the scope and limitations of the MUA procedure, indicating that it was not a panacea for low back pain [14].

As the fundamental basis for SMUA, the adhesion-disruption theory remains clinically appealing. The term Fibrosis Release Procedures (FRP) is a broader designation for spinal manipulation under anesthesia which has been more recently introduced in the chiropractic literature and within the published AAMUAP guidelines [3]. This terminology establishes that mobilization (stretching) is the primary manual therapy component of the procedure.

The premise that reducing adhesions can increase joint flexibility, decrease pain, and improve quality of life is seemingly so elementary that utilizing approaches which aim to accomplish the like must be therapeutically beneficial. There is evidence, for example, that MUA for stiff knees after total knee arthroplasty (TKA) can significantly increase knee flexion in certain patients when administered during the postoperative period but within twelve weeks [18]. The circumstances by which adhesions may develop after knee replacement are self-evident, giving due consideration to the mode and scope of intervention. The proliferation of scar tissue about the knee joint is a well-known complication of TKA [19]. However, that clinical scenario differs greatly from one that involves the presence of chronically symptomatic and dysfunctional spinal joints due to repetitive postural strain, factors related to age, or a history of trauma that excludes vertebral fracture or reconstructive surgery after disc injury. Thus, the evidence that MUA may reduce TKA-related arthrofibrosis is not generalizable to chronic spine pain patients who have been managed non-surgically for lower-level injury or degenerative change. In the latter patient populations, the supposition that scar tissue explains any observed range of motion deficits may be presumptuous.

Investigators have recognized that patient selection for SMUA could be enhanced by knowing more about the fibrotic adhesion concept [20]. One theory concerning adhesion development suggests that lumbar spine hypomobility may result in connective tissue adhesions of zygapophyseal joints [21]. While increased collagen deposition about the spine could explain the physical findings of palpable joint restrictions and decreased range of motion in chronic pain patients, there is no research that may assist in differentiating those with intraarticular adhesions from other manifestations of segmental dysfunction. In fact, validation of the adhesion concept and a related clinical role for SMUA would appear to have its greatest potential in cases of failed back surgery, in which advanced imaging reveals fibrosis in the region of prior intervention [16]. It has been estimated that as much as 20% to 36% of all cases of failed back surgery may be due to epidural fibrosis [22]. Nevertheless, for post-fusion lumbar pain there is limited evidence for the safety and efficacy of chiropractic treatment and no guidelines to aid with therapeutic decision-making [23].

Clinical research is lacking to support that the many historical accounts of successful SMUA treatment is due to the disruption of ligamentous, musculotendinous and/or epidural adhesions. The void of evidence in this area does not allow one to confirm or deny the theories that SMUA more effectively treats adhesions and that adhesion reduction increases flexibility [1]. With various spinal tissues known to account for pain and impaired function, it defies clinical logic that adhesions might serve as a pathological feature common to patients with chronic pain. Thus, what may account for a favorable response to SMUA treatment, and purportedly for numerous diagnoses [3], has yet to be elucidated. The genetic profiles that contribute to fibrosis and ligament hypertrophy in specific spinal conditions [24, 25], and how identifying the like may assist in patient selection/rejection for SMUA, are areas worthy of investigation. Nevertheless, recent evidence suggests that stretch does not have significant effects on joint mobility for people with or without neurological conditions, or short-term effects on pain or quality of life for people with non-neurological conditions [26].

## Predictors of unfavorable clinical outcomes with SMUA

In the development of treatment protocols for SMUA professional associations have relied upon numerous studies from the early medical literature. Advocates for the procedure frequently cite these same studies to support the use of SMUA for various diagnoses. In the more recent literature are reports of the indications for manipulation under anesthesia, including disc herniation/prolapse/protrusion/bulge, joint or spinal ankylosis, failed low back surgery, nonresponsive muscle contraction, compression syndromes with non-osteophytic entrapment, and whiplash-associated disorders [27, 28]. Most of these indications for care have been derived from the syllabi of chiropractic post-graduate MUA certification courses and the promotional materials of MUA proponents. However, numerous clinically significant findings from the same dependent literature base have yet to be given an appropriate level of attention. Several clinical, diagnostic, and litigation-related indices must now be recognized as predictors of unfavorable clinical outcomes or for potential adverse events. These indices, identified below, do not support the preservation of certain patient management concepts and selection criteria for SMUA, as initially developed before the era of evidence-based medicine.

### Ankylosis

Spinal ankylosis has been cited as one of the indications for SMUA [27, 28]. However, when joints are pathologically fused, normal physiologic integrity or function cannot be expected to be restored via the stretching and/or manipulation components of the procedure. This requires no analysis. A case involving spine fracture and hemothorax with MUA treatment for ankylosing spondylitis has been reported [13].

### Anxiety/stress

The prospective cohort study undertaken by Peterson et al. (Level II evidence) suggests a contributory role of anxiety and stress levels on outcome for patients in receipt of SMUA for chronic neck or low back pain [29]. When comparing short-term patient outcomes after a single MUA procedure dose (at 2 and 4 weeks posttreatment), statistically significant differences were found for those improved vs not improved when assessing for the variable of anxiety/stress [29]. Patients with higher levels of anxiety and stress, identified by the Bournemouth Questionnaire (BQ), had a tendency of non-responsiveness to SMUA [29].

### Electromyography and nerve conduction studies

Published MUA guidelines specify that electromyography (EMG) and/or nerve conduction studies may be used to determine patient progress with SMUA [3]. Implied, in part, is that a positive EMG study may serve as a diagnostic indicator for treatment. For years, MUA advocates have put forth that the work of Mensor showed that 83% of 600 patients with EMG-verified radiculopathy responded well to SMUA [30]. However, within Mensor’s published papers, there is no reporting of an electromyographic assessment for any of the more than 600 patients treated with SMUA [31, 32]. In fact, from the findings of one of the few studies that reaches the highest level of published evidence to date (Level II), SMUA in the presence of EMG-confirmed lumbar nerve root compression led Siehl et al. to report an outcome trend of procedural ineffectiveness, with surgery likely required at some point [33]. Thus, current best evidence does not support the premise that patients with a positive EMG study are appropriate candidates for SMUA. As for nerve conduction studies, there is no primary research evidence that may be assistive to clinicians in revealing the appropriateness of SMUA for individual patients with positive versus negative results. Moreover, the potential clinical value for using either of these electrodiagnostic tests in an outcomes assessment capacity with SMUA is not supported by research.

### Herniated and/or protruding/bulging discs

Greenman cited disc herniation as a relative contraindication to MUA, acknowledging that other authors had reported only temporary improvement [34]. In looking to the evidence for long-term effectiveness and safety of SMUA with a specific diagnosis of disc herniation or protrusion, Table 1 provides the findings, observations, or experience-based perspectives of numerous early osteopathic/orthopedic investigators/practitioners.

**Table 1:** Evidence of outcomes with SMUA for disc herniation/protrusion

Year of Publication	Author/s	Findings/observations/opinions	Level of Research Evidence^a^
1945	Poppen [11]	Various forms of operative treatment were undertaken for 400 cases of lumbar intervertebral disc herniation. The number of patients whose treatment included MUA is not reported, but two from that group were immediately paralyzed.	IV
1952	Wilson and Ilfeld [78]	Manipulation of patients with symptoms of lumbar herniated disc was performed under general anesthetic or via medication assistance. Three of eighteen patients (17%) reported temporary relief of back and leg pain over 48–72 h. Within ten days of the procedure, twelve patients (67%) subsequently underwent exploratory laminectomy. For the remaining six patients (33%) who did not undergo surgery, none had experienced any change in symptoms after manipulation.	IV
1952	Siehl and Bradford [79]	Good results were obtained in about a third of herniated disc cases but with surgery opined to be required at some point. The authors reported that longstanding disc herniation does not respond well to MUA and, “in no case with positive myelography has there been lasting good results from the manipulative procedure.”	IV
1953	Ewer [80]	Manipulation should not be overlooked by orthopedic surgeons in that it can offer “so much relief” in selected cases. However, it was opined that with ruptured intervertebral discs and true sciatica, manipulation “cannot effect a permanent cure and offers great hazards.” In the presence of space-consuming lesions manipulation is contraindicated, as MUA “is more dangerous and does not compensate for the risks involved.”	V
1955	Mensor [31]	Two hundred five patients received MUA for lumbar intervertebral disc syndrome, with 56 (27%) classified as immediate or delayed failures. Of those, subsequent surgical exploration revealed that all had identifiable pathology (an annular fragment protruding into the interspace, a ruptured annulus with a large amount of free nuclear material in the canal, or degeneration with freely shifting nuclear material permitting for alternating reduction and reproduction of the protrusion).	IV
1963	Siehl [81]	One hundred eighty five patients were treated with MUA for a diagnosis of herniated nucleus pulposus. Good results were obtained for 26.4% of patients. Overall, 95 of the 185 patients (51%) required subsequent disc surgery.	IV
1964	Chrisman et al. [15]	Twenty of 39 patients (51%) with ruptured lumbar intervertebral disc maintained good to excellent results after MUA over three years. 10 of the 27 patients (37%) with positive myelograms had received the same benefit. For neither group was there a change in the appearance of the myelograms taken before and after MUA. The authors determined that those “without a demonstrable myelographic defect consistently did better” and that manipulation of “a very large disc protrusion” should be avoided due to potential for harm.	IV
1971	Siehl et al. [33]	Twenty one patients were treated via MUA for nerve root compression secondary to lumbar disc herniation. Three (14%) showed clinical and EMG improvement, nine (43%) had no EMG change but continued clinical improvement, and nine (43%) showed worsened electromyographic changes of the legs. After 15 months, the latter group had an increase in clinical signs. In general, for the 50% of patients who had improvement over the first 6 months, most progressively worsened over the 6 month period thereafter.	II
1972	Tospon [82]	In the author’s experience with 6000 MUA procedures, he reported, “.if the patient has positive neurological, orthopedic and myelographic findings, low back manipulation will be of no lasting benefit... it often helps temporarily, but ultimately surgery has to be performed.”	V^b^
1973	Morey [83]	“Frequently it [manipulation under general anesthesia] affords relief, possibly temporary, when there is actual disk protrusion.”	IV
1977	Scherrer [84]	Ninety four patients underwent manipulation under general anesthesia for disc herniation. Sixty percent had excellent or good results. Forty percent had poor results. Within one year, more than half of the patients had to undergo a hemilaminotomy.	IV
1986	Krumhansl and Nowacek [47]	Of the two patients with myelogram evidence of frank disc herniation, one required discectomy because of a return of pain within three weeks of MUA.	IV

^a^When applying the levels of evidence rating system for categorizing study quality, as put forth by Wright et al. and adopted by the *Journal of Bone & Joint Surgery* [42], *Spine, Clinical Orthopaedics and Related Research*, the North American Spine Society, the American Academy of Orthopaedic Surgeons, and the Pediatric Orthopaedic Society of North America [85]

^b^The case report study design has not been rated by Wright et al. [42]. This case report, with editorializing, is being equated here with the established level of evidence for expert opinion (Level V evidence)

There is no published evidence to suggest that the modern SMUA approach [1] provides for better outcomes for disc herniation/protrusion versus the methods and protocols used by early osteopathic investigators. Within the more recent chiropractic literature there are only a few isolated retrospective case reports regarding the sedated variety of SMUA for disc herniation [17], protrusion/bulge [16, 35, 36], or degeneration [37]. In the study conducted by Palmieri and Smoyak, some of the 38 chronic low back pain patients who received MUA may have had a lumbar disc condition [38]. However, the various causes of pain, which included “disc syndrome”, were apparently obtained by demographic questionnaire. There is no account of investigator verification of the cause of back pain via clinical examination or imaging. Moreover, as the decreased pain and disability scores with MUA are not correlated with an identified cause of pain, the outcomes for the “disc syndrome” category of patients are not evident. Elsewhere, for the 42 chronic low back pain patients who received medication-assisted manipulation after MRI, the presence or absence of disc pathology is not reported in revealing the nature of the conditions under study [39]. For the 30 chronic neck or low back pain patients in receipt of SMUA in the prospective cohort study undertaken by Peterson et al., there is no indication of a verified source of pain [29].

**Fig. 1:**
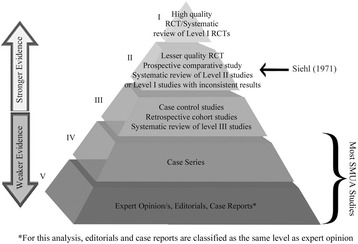
SMUA evidence for disc herniation/protrusion

Within the chiropractic literature is a description of the need for provider modification or deletion of the maneuvers typically used with SMUA when disc herniation is present [36]. However, clinical investigation is lacking to support the safety and efficacy of any particular intraoperative MUA technique. Also, it remains unclear as to whether the size and direction of disc protrusion, the spinal region involved, the location and extent of annular tearing, the degree of disc degeneration/desiccation, or the extent and duration of correlating clinical signs and symptoms may serve as prognosticators of outcome with SMUA. With *conscious* patients, there have been reports of favorable outcomes with the utilization of selective spinal manipulation procedures/techniques for specific MRI-confirmed lumbar disc herniation features or positional variances [40, 41]. However, in these studies it is not clear that either of the two manipulative techniques described, chosen based on the particular imaging feature/s, was superior to the other such that its use specifically explained the improvements reported [40, 41]. Additional research is needed to clarify which of the aforementioned elements, or other discopathy-related variables, may have predictive value in the SMUA setting. Presently, the evidence for SMUA for disc-related conditions remains weak (mostly Level IV-V), with overall unimpressive percentages of successful cases reported by early authors and across larger samples of subjects. By way of the research evidence hierarchy put forth by Wright et al. [42], Fig. 1 outlines the current state of the evidence for SMUA for disc herniation/protrusion.

With potential for adverse events, the equivocal benefit profile of SMUA in the presence of disc pathology reveals that it has yet to attain precise clinical validation through investigative research. In the *conscious* patient setting, the North American Spine Society has found insufficient evidence to recommend for or against manipulation for lumbar disc herniation with radiculopathy, as an alternative to discectomy or to improve functional outcomes [43]. Nonetheless, the emergence of newer, preliminary evidence may suggest the efficacy of spinal manipulation in *conscious* patients with MRI-confirmed symptomatic lumbar herniated discs with varied pathoanatomical features, including sequestration [41]. Similar evidence of efficacy exists for spinal manipulation in *conscious* patients with symptomatic, MRI-confirmed cervical disc herniations [44]. What remains unclear is whether the addition of anesthesia to the manipulation service, along with the SMUA-associated maneuvers/techniques, may result in better outcomes over manipulation alone for acute or chronic neck and low back pain due to disc herniation/protrusion. That was not found to be true in the comparative studies for subjects with a diagnosis of chronic lumbosacral strain [45, 46]. Thus, precaution must be taken when contemplating SMUA for those with verified or suspected disc-related conditions. Greater weight should first be given to other, well-established interventions.

### Obesity and soft tissue inflammation

Krumhansl and Nowacek reported that patients who are overweight by greater than 50 pounds are not candidates for SMUA, as the “procedure fails” due to the diffusion of force within soft tissues [47]. Also reported was “a much reduced success rate” with patients who are overweight by greater than 30 pounds. For similar reasons of impeded maneuverability of spinal tissues, these practitioners cited an absolute contraindication for patients who exhibit soft tissue inflammation beyond a “mild” degree (greater than 2+, on the scale of 0–4+). A “very high” failure rate was reported for inflammation beyond grade 2+, with risk for injury to uninvolved soft tissues placed under the stresses of manual therapy [47]. Thus, as described by these authors, the physiologic indicators of obesity and inflammation weigh against the option of SMUA treatment.

### Work-related spine disorders and litigation

Mensor reported a significantly lower percentage of favorable patient outcomes with MUA for industry-related lower back conditions versus “private patients” with similar conditions [31]. He attributed this outcome discrepancy to “the monetary factor” and the type of labor engaged in by those in the industrial patient group [31]. Similarly, Chrisman et al. observed comparatively poorer outcomes for patients in receipt of SMUA for lumbar conditions who were involved in litigation or had compensation claims [15]. In one of the few SMUA studies to date that represents Level II evidence, Kohlbeck et al. excluded patient participation if chronic low back pain was associated with third-party liability or workers’ compensation [39].

Barth has suggested that examinee-reported histories are largely false when someone other than the examinee is being blamed for the health complaints [48]. Also cited by Barth are the influences of psychological and social factors in chronic pain presentations, especially with legal claim involvement [49]. The variable of secondary gain that may result in chronic pain, poor patient outcomes, or invalid patient reporting [49–51] are not limited to those being considered for SMUA. However, as SMUA utilization is closely associated with chronicity after personal injury, consideration must be given to prior investigator outcomes reporting for cases involving litigation. In the presence of additional identifiable indices for unfavorable/adverse outcomes, the option of SMUA treatment may not be appropriate for individual patients with a legal claim.

## Pain prone patients and candidacy for SMUA

In determining a patient’s candidacy for chiropractic treatment of an acute or chronic musculoskeletal condition, many core principles apply. These are not unique to clinical decision-making for SMUA. However, the same obligation exists for SMUA as with any modality or intervention being considered. For acute, non-specific low back pain, clinical practice guidelines consistently recommend patient reassurance of a good prognosis and educational instruction on self-care and remaining active [52]. Trajectory patterns for low back pain reveal that acute pain is often an episode or a flare-up of an ongoing condition, but pain in excess of three months would suggest a very different condition [53]. Once chronic, low back pain can be more difficult to manage and may require multimodal care [54]. Also, in the presence of widespread pain, chronic non-specific low back pain has a poorer prognosis than if pain is present only in the lower back [55].

Poor outcomes with conventional conservative treatment can often be predicted. It is the provider’s duty to identify potential barriers to recovery. For example, Bigos et al. identified premorbid nonphysical factors such as job dissatisfaction to be the best predictor of reporting back pain at work [56]. Also, clinical prediction/decision rules have been proposed for patient responsiveness to lumbar stabilization exercises [57] and those at risk for developing chronic low back pain [58]. In these and other instances, use of clinical prediction rules may help to determine the most appropriate course of treatment or if manipulation should even be considered [59]. Attention to the patient history is particularly important, as the reliability of the information gleaned may be questionable when litigation potential or secondary gain are present [60, 61].

Absent from the MUA literature is insight regarding the significance of the patient’s attitudes, beliefs, and expectations of treatment. Factors such as secondary gain, somatization, illness-behavior, and physician dependence are essentially without mention, yet represent key components to the management of chronic musculoskeletal pain [54, 62]. Moreover, although it is known that behavioral treatment can prevent chronicity [55], related elements such as alcohol/illicit drug use may be overlooked during the patient history and ensuing course of care. In clinical and especially medicolegal circumstances, embellishment and symptom magnification are not uncommon [62]. If not recognized and addressed early on, these factors could affect the direction and outcome of care. Thus, a thorough clinical examination, with attention for non-physiologic responses, is paramount. Still bearing relevance today is this AHCPR appraisal on pain behavior and inconsistent findings:

“The patient who embellishes a medical history, exaggerates pain drawings, or provides responses on physical examination inconsistent with known physiology can be particularly challenging. A strongly positive supine straight leg raising test without complaint on sitting knee extension and inconsistent responses on examination raise a suspicion that nonphysical factors may be affecting the patient's responses. "Pain behaviors" (verbal or nonverbal communication of distress or suffering) such as amplified grimacing, distorted gait or posture, moaning, and rubbing of painful body parts may also cloud medical issues and even evoke angry responses from the clinician. Interpreting inconsistencies or pain behaviors as malingering does not benefit the patient or the clinician. It is more useful to view such behavior and inconsistencies as the patient's attempt to enlist the practitioner as an advocate, a plea for help. The patient could be trapped in a job where activity requirements are unrealistic relative to the person's age or health. In some cases, the patient may be negotiating with an insurer or be involved in legal actions. In patients with recurrent back problems, inconsistencies and amplifications may simply be habits learned during previous medical evaluations. In working with these patients, the clinician should attempt to identify any psychological or socioeconomic pressures that might be influenced in a positive manner. The overall goal should always be to facilitate the patient's recovery and avoid the development of chronic low back disability.” [63]

One of several concerns with SMUA utilization relates to proper patient selection beyond the factors of physical complaints and findings. This may be of greatest relevance in the personal injury setting, since patients within that setting represent the preponderance of SMUA recipients in the United States today. On the historical basis that spinal manipulation may be the “treatment of choice,” MUA proponents have considered SMUA to be the logical next step for chronic pain patients with limited response. Nonetheless, not all patients can be expected to respond to the treatment chosen by the attending provider. Furthermore, failure of one option to provide for measurable and objective clinical gains, such as conventional office-based manipulation, would rarely support advancing the patient to the SMUA setting. Numerous conservative treatment options exist. Those with more robust evidence should be considered first, as dictated by the best-interest principle [54].

Another concern relates to the circumstance of offering the option of SMUA relatively early in care. This likely occurs more frequently than it should despite that the procedure lies low in the hierarchy of evidence-based treatment. As early patient advancement to SMUA stands apart from the existing literature on patient management, additional risk/benefit issues arise. The concepts that hurt isn’t always harm and that some patients get better with or without treatment must be given adequate consideration relative to the natural course of an injury. An individual who is likely to demonstrate additional improvement based on time alone may have no need for exposure to the added risk of medication assisted manipulation, even if that risk is minor. At times, a particular patient may be predestined to receive a full complement of therapeutic services and diagnostic tests early on, only to be followed by a treatment option of “last resort” if symptoms remain after a few weeks. But with that is the failure to incorporate unique anatomic, physiologic, and psychosocial variables in clinical decision making. Setting the individual patient’s expectations that the only way to improve is by way of treatment “x” undermines both the contemporaneous treatment being pursued and the available alternatives. Under these circumstances, the patient preference/value component of the evidence-based practice model [54] becomes prone to the influences of provider habit, preference, and assuredness for treatment “x”.

## Defining levels of risk and informed consent with SMUA

In the office setting, the risks associated with spinal manipulative therapy (SMT) are relatively low as compared with common modes of medical treatment [54]. However, a recent systematic review found inadequate reporting of adverse events with spinal manipulative therapy (SMT), whether catastrophic or otherwise [64]. Swait and Finch report that serious adverse events with manual treatment of the spine appear to be rare, making it difficult to estimate risk level [65]. They note that pre-existing pathology may increase the risk of some events and call for enhanced knowledge through clinician use of patient safety incident reporting systems [65]. Cassidy et al. reported no evidence of increased risk of vertebrobasilar artery (VBA) stroke [66] or carotid artery stroke [67] associated with chiropractic treatment versus primary care, suggesting coincidental occurrence. Thus, the possibility exists for a cervical arterial stroke to be the cause of neck pain rather than the result of manual intervention [65–67]. Although it has been proposed that neck hyperextension during intubation may lead to vertebral artery dissection (VAD) [68], any specific association between VAD and vascular mechanical stress during neck manipulation under anesthesia has yet to be studied. Cadaveric studies have shown that chiropractic manipulation of the neck does not cause vertebral arterial strain or internal carotid arterial strain in excess of the strains incurred with ordinary movements [69, 70]. As for lumbopelvic spine manipulation-related cauda equina syndrome (CES), a recent systematic review cites it as the most common of the serious adverse events reported but excludes the assessment of cases involving manipulated under anesthesia [71]. Nevertheless, manipulating the lumbar spine under anesthesia may present an increased risk for CES over manipulation alone, as per anecdotal reporting that sixteen of twenty-nine identified CES cases involved manipulation under narcosis or ether anesthesia [72].

Medical malpractice carrier statistics on adverse events with MUA are proprietary and unavailable for analysis in the public domain. The SMUA literature contains mostly retrospective case reports and case series with selective focus on the benefits of treatment. Thus, the incidence for failed treatment or adverse events cannot be determined. With the rate of minor to serious complications remaining unknown, better case reporting and investigative efforts on the safety profile of SMUA are needed. For the chiropractic clinician who may not be particularly expert on certain aspects of the individual patient’s medical history, concurrent conditions, and prescription/illicit drug use, clinical decision making for SMUA should be reserved until all appropriate consultations are pursued. Any area of concern or uncertainty identified should result in a referral to a specialist who has the needed expertise to determine risk level and is best positioned to provide for medical clearance.

Spinal manipulation under anesthesia brings additional risk exposure beyond office-based conscious manipulation. Prior to anesthetization, it is incumbent upon the chiropractor to be aware of these risks and overall patient fitness for SMUA. For example, it is known that poor general health increases the risks inherent to anesthesia. General health risk categories for anesthesia have been established by the American Society of Anesthesiologists (ASA) [73], with which SMUA providers must be familiar when directing patients through pre-procedure screening evaluations. Also, consideration must be given by the chiropractor to the risks and contraindications for the manual therapy component of the MUA procedure. These expand from those associated with office-based manipulation. As mitigation and outcomes optimization strategies for the manual therapy component of the MUA procedure, Table 2 provides examples of clinical predictors of risk and outcome potential. The elements therein have been placed on a scale similar to that established by the ASA for predictors of perioperative mortality rates, and as adopted by the American College of Physicians for predictors of postoperative pulmonary and cardiac complications [74]. As with the ASA Physical Status Classification System, it has been shown that offering examples for each category aids with proper patient assignment [75].

**Table 2:** Manual therapy risk/outcome stratification with SMUA

SMUA Classification	Patient profile or status	Examples^b^
SMUA I	A normal, healthy patient with no frank clinical predictor for unfavorable outcome or harm	A 40 year old male with a prior history of repetitive sports-related trauma to the low back, and: - a normal neurological examination - MRI findings of mild multilevel lumbar disc degeneration - a more recent history of chronic recurrent back pain with significant debilitation, active/passive range of motion deficits, and muscle guarding - limited response to adequate trials of office-based thrust manipulation and other modes of conservative care
SMUA II	An otherwise normal, healthy patient with a clinical predictor or profile for unfavorable outcome but not harm	Obesity, high anxiety/stress level^a^, litigation, work-related injury, somatizer, significantly inadequate response to an office-based trial of treatment including thrust manipulation
SMUA III ^c^	A patient with identifiable signs, symptoms or a history of comorbidity that may predict harm despite potential for diminution/remediation of complaint	HTN Stage 1^e^, DVT, acute or chronic respiratory condition, history suggestive of osteoporosis, current history of drug or alcohol abuse, cancer history, night pain, unintentional weight loss, unexplained dizziness, structural deformity, ligamentum flavum hypertrophy, lumbar disc herniation/protrusion, positive lumbar EMG, corticosteroid use, prior non-fusion surgery to site of treatment, spinal fusion with adjacent segment disease, worsening of symptoms with office-based thrust manipulation
SMUA IV^d^	A patient with significant comorbidity or a history that likely predicts unfavorable outcome and/or potential for harm	HTN Stage 2^f^, angina pectoris, unstable bleeding disorders, uncontrolled diabetes, pain prone patient, hysteria, inflamed spinal tissues [47], joint hypermobility/instability, unstable spondylolisthesis, severe joint sprain, joint dislocation, advanced spondylosis with osteophytosis of or about the spinal canal or IVF, implant instrumentation (precludes manipulation to the site), advanced osteoporosis, multiple myeloma, joint/bone infection, acute inflammatory arthritis/gout, positive myelogram, marked motor signs, diastematomyelia, positive plantar reflex/clonus, ataxia
SMUA V^d^	A patient with a highly significant clinical condition or comorbidity that readily predicts unfavorable outcome, harm or death	Hypertensive Crisis^g^, advanced carotid/vertebral artery disease, unstable aneurysm, acute abdominal pain with guarding, intracranial/intracanalicular hematoma, recent fracture, ankylosing spondylitis, malignant bone tumor or metastatic disease to bone, aggressive benign bone tumor, Paget’s disease, Tuberculosis of bone, disc sequestration, Arnold Chiari malformation, spinal cord/meningeal tumor, Cauda Equina Syndrome, bladder dysfunction, saddle anesthesia, myelopathy, septicemia, known anesthesia allergy

^a^When assessed by Bournemouth Questionnaire, as per the findings of a recent prospective cohort study (Level II evidence) [29]

^b^Examples include identifiable factors of unfavorable outcome, as reported by prior investigators, as well as relative and absolute contraindications to manipulation of unconscious patients. Contraindications and/or potential exclusion criteria are not limited to those shown

^c^Modification of technique may be required with this risk category, assuming that medical clearance has been obtained via sufficient multidisciplinary input for the specific precaution/s for harm. Examples of proper specialty input include a cardiologist for HTN Stage 1, and a vascular surgeon for DVT

^d^This category represents a red flag classification for the SMUA service

^e^Systolic mm Hg of 130–139 ***or*** diastolic mm Hg of 80–89 [86]

^f^Systolic mm Hg of 140 or higher ***or*** diastolic mm Hg of 90 or higher [86]

^g^Systolic mm Hg higher than 180 ***or*** diastolic mm Hg higher than 120 [86]

As noted across the early SMUA publications and within the consensus statement put forth by the American Academy of Osteopathy (AAO), the single-dose approach is most common [9]. Under AAO criteria, if a second procedure dose is to be considered, it is usually after a three-week period [76]. Such would permit sufficient time to gauge the outcome of the initial procedure and to determine if it was sufficiently restorative in scope to represent clinical endpoint. With the SMUA approach put forth by the AAMUAP, it has been proposed that customary use of serial dosing provides for increased safety through gentler treatment (with “better control of biomechanical force,” when administered over three consecutive days) [3]. Nevertheless, each anesthesia exposure event would carry its own separate risks, whether inherent to the anesthetic agent or for the successive encounters during which the unconscious patient is unable to alert the provider to a painful and potentially injurious maneuver.

The ambulatory surgical center setting (ASC) in which SMUA is typically performed presents its own risk concerns. First, it must be established that patients are free from mental impairment and have an intellectual status permitting for adherence to ASC-related pre-procedural and post-procedural instructions. Second, it is required that patients have social support by way of a companion for transportation/observation, in adhering to procedural logistics and the at-home recovery period following ASC discharge. Last, because the ASC simply does not have the operational depth to meet that of a large medical center, it is not as well positioned to manage patients with poorly compensated or incompletely evaluated systemic disease, issues of acute substance abuse, an abnormal airway predisposing to difficult intubation, or patients having a personal or strong family history of anesthesia-associated malignant hyperthermia.

SMUA is an elective procedure. As a treatment option for a non-life threatening disorder, it is scheduled in advance. This provides adequate time for informed consent and/or patient pursuit of a second opinion from a specialist not involved in MUA. As put forth by Globe et al. informed consent is defined as a communication process between doctor and patient that leads to the patient agreeing to undergo a specific intervention [54]. Material risks of the intervention and available treatment options should be explained to the patient, along with the risks of no treatment [54]. It is the responsibility of the doctor to disclose information that is known and that the patient would find important in deciding whether to undergo a particular procedure [77]. Clinicians who perform spinal manipulation under anesthesia, or make referrals for the like, are obligated to understand the state of the evidence, procedural contraindications/risks, and the conditions and indicators that are predictive of adverse or unfavorable outcomes. As such, that information may be communicated to patients as they contemplate which of the available treatment options to pursue.

## Conclusion

This paper adds to the body of knowledge for SMUA by evaluating the existing evidence in a unique way. It addresses outcome potential in several areas, identifies prognostic factors for complication, and qualifies where primary research efforts are needed for improved patient selection. As a primer, a new risk classification system is introduced which may serve as a guide for use in clinical practice. No longer can treatment standards be reliant upon anecdotal reports of satisfied patients, proclamations of the various conditions amenable to SMUA, preconceptions of permanent therapeutic benefit, a philosophy that calls for serial and multiregional treatment applications, and/or the fact that the procedure has had a historically good safety record. As health care professionals committed to the public interest, chiropractors who perform or make referrals for SMUA are obligated to remain current and put forth accurate information in the public domain on the state of the evidence, known procedural contraindications/risks, and the conditions and indicators that may predict an adverse or unfavorable clinical outcome. When chiropractors lack a comprehensive understanding of that information, or advocate for the procedure by preference, patients may be unable to make informed decisions about their health care options in submitting to appropriate consent. With a new perspective on the evidence for SMUA, this analysis can assist clinicians who are seeking to identify key decision points in care in enhancing risk management strategies and optimizing patient outcomes.

## Abbreviations

CES: Cauda equina syndrome
EMG: Electromyography
FRP: Fibrosis Release Procedures
SMT: Spinal manipulative therapy
SMUA: Spinal manipulation under anesthesia
VAD: Vertebral artery dissection
VBA: Vertebrobasilar accident

## References

[1] Dagenais S, Mayer J, Wooley JR, Haldeman S (2008). Evidence-informed management of chronic low back pain with medicine-assisted manipulation. Spine J.

[2] Gordon RC (2001). An evaluation of the experimental and investigational status and clinical validity of manipulation of patients under anesthesia: a contemporary opinion. J Manip Physiol Ther.

[3] Gordon R, Cremata E, Hawk C (2014). Guidelines for the practice and performance of manipulation under anesthesia. Chiropr Man Therap.

[4] Jackson R, Feder G (1998). Guidelines for clinical guidelines. BMJ.

[5] Cates JR, Young DN, Bowerman DS, Porter RC (2006). An independent AGREE evaluation of the occupational medicine practice guidelines. Spine J.

[6] Cates JR, Young DN, Guerriero DJ, Jahn WT, Armine JP, Korbett AB, Bowerman DS, Porter RC, Sandman T, King RA (2003). An independent assessment of chiropractic practice guidelines. J Manip Physiol Ther.

[7] Cates JR, Young DN, Guerriero DJ, Jahn WT, Armine JP, Korbett AB, Bowerman DS, Porter RC, Sandman TD, King RA (2002). Independent guideline appraisal summary report for vertebral subluxation in chiropractic practice (CCP) guidelines. J Chiropr Med..

[8] Field MJ, Lohr KN (1990). Clinical practice guidelines: directions for a new program. Institute of Medicine (US) committee to advise the public health service on clinical practice guidelines.

[9] DiGiorgi D. Spinal manipulation under anesthesia: a narrative review of the literature and commentary. Chiropractic Man Ther. 2013;21(1):14. 10.1186/2045-709X-21-14 PMC3691523 23672974

[10] Pauker SG, Kassirer JP (1975). Therapeutic decision making: a cost-benefit analysis. N Engl J Med.

[11] Poppen JL (1945). The herniated intervertebral disk- an analysis of 400 verified cases. NEJM.

[12] LaMendola B. Medical safety spotlight growing- man unresponsive after ‘manipulation under anesthesia: March 22, 2009. Sun Sentinel. http://articles.sun-sentinel.com/2009-03-22/news/0903210114_1_mua-chiropractor-procedure. Accessed 17 Nov 2017

[13] Gardner SC, Majercik SD (2013). Man, 57, with Dyspnea after chiropractic manipulation. Clin Rev.

[14] Clybourne HE (1948). Manipulation of the low back region under anesthesia. J Am Osteopath Assoc..

[15] Chrisman OD, Mittnacht A, Snook GA (1964). A study of the results following Rotatory manipulation in the lumbar Intervertebral-disc syndrome. J Bone Joint Surg Am.

[16] Davis CG, Fernando CA, da Motta MA (1993). Manipulation of the low back under general anesthesia: case studies and discussion. Journal of the Neuromusculoskeletal System..

[17] Herzog J. Use of cervical spine manipulation under anesthesia for management of cervical disk herniation, cervical radiculopathy, and associated cervicogenic headache syndrome. J Manipulative Physiol Ther. 1999;22(3):166–70. 10.1016/S0161-4754(99)70131-4 10220716

[18] Vanlommel L, Luyckx T, Vercruysse G, Bellemans J, Vandenneucker H. Predictors of outcome after manipulation under anaesthesia in patients with a stiff total knee arthroplasty. Knee Surg Sports Traumatol Arthrosc. 2017;25(11):3637–3643. 10.1007/s00167-016-4413-6 28032122

[19] Cheuy VA, JRH F, Paxton RJ, Bade MJ, Zeni JA, Stevens-Lapsley JE (2017). Arthrofibrosis associated with Total knee Arthroplasty. J Arthroplast.

[20] Morningstar MW, Strauchman MN (2012). Manipulation under anesthesia for patients with failed back surgery: retrospective report of 3 cases with 1-year follow-up. J Chiropr Med..

[21] Cramer GD, Henderson CN, Little JW, Daley C, Grieve TJ (2010). Zygapophyseal joint adhesions after induced hypomobility. J Manip Physiol Ther.

[22] Epter RS, Helm S, Hayek SM, Benyamin RM, Smith HS, Abdi S (2009). Systematic review of percutaneous adhesiolysis and management of chronic low back pain in post lumbar surgery syndrome. Pain Physician.

[23] Daniels CJ, Wakefield PJ, Bub GA, Toombs JD (2016). A narrative review of lumbar fusion surgery with relevance to chiropractic practice. J Chiropr Med..

[24] Sun C, Tian J, Liu X, Guan G (2017). MiR-21 promotes fibrosis and hypertrophy of ligamentum flavum in lumbar spinal canal stenosis by activating IL-6 expression. Biochem Biophys Res Commun.

[25] Xu YQ, Zhang ZH, Zheng YF, Feng SQ (2016). MicroRNA-221 regulates hypertrophy of Ligamentum Flavum in lumbar spinal Stenosis by targeting TIMP-2. Spine.

[26] Harvey LA, Katalinic OM, Herbert RD, Moseley AM, Lannin NA, Schurr K (2017). Stretch for the treatment and prevention of contractures. Cochrane Database Syst Rev.

[27] Morningstar MW, Strauchman MN (2010). Management of a 59-year-old female patient with adult degenerative scoliosis using manipulation under anesthesia. J Chiropr Med..

[28] Taber DJ, James GD, Jacon A (2014). Manipulation under anesthesia for lumbopelvic pain: a retrospective review of 18 cases. J Chiropr Med.

[29] Peterson CK, Humphreys BK, Vollenweider R, Kressig M, Nussbaumer R (2014). Outcomes for chronic neck and low back pain patients after manipulation under anesthesia: a prospective cohort study. J Manip Physiol Ther.

[30] International MUA. Academy of physicians: an overview of manipulation under anesthesia (MUA). Taylor & Francis Group. 2005; http://muaphysicians.com/overview.html. Accessed 17 Nov 2017

[31] Mensor MC (1955). Non-operative treatment, including manipulation, for lumbar intervertebral disc syndrome. J Bone J Surg.

[32] Mensor MC (1965). Non-operative treatment, including manipulation, for lumbar intervertebral-disc syndrome. J Bone Joint Surg Am.

[33] Siehl D, Olson DR, Ross HE, Rockwood EE. Manipulation of the lumbar spine with the patient under general anesthesia: evaluation by electromyography and clinical-neurologic examination of its use for lumbar nerve root compression syndrome. J Am Osteopath Assoc. 1971;70:433-440. 5203536

[34] Greenman PE (1992). Manipulation with the patient under anesthesia. J Am Osteopath Assoc..

[35] Hughes BL (1993). Management of cervical disk syndrome utilizing manipulation under anesthesia. J Manip Physiol Ther.

[36] Cremata E, Collins S, Clauson W, Solinger AB, Roberts ES (2005). Manipulation under anesthesia: a report of four cases. J Manip Physiol Ther.

[37] Davis CG (1996). Chronic cervical spine pain treated with manipulation under anesthesia. Journal of the Neuromusculoskeletal System.

[38] Palmieri NF, Smoyak S (2002). Chronic low back pain: a study of the effects of manipulation under anesthesia. J Manip Physiol Ther.

[39] Kohlbeck FJ, Haldeman S, Hurwitz EL, Dagenais S (2005). Supplemental care with medication-assisted manipulation versus spinal manipulation therapy alone for patients with chronic low back pain. J Manip Physiol Ther.

[40] Peterson CK, Leemann S, Lechmann M, Pfirrmann CW, Hodler J, Humphreys BK (2013). Symptomatic magnetic resonance imaging-confirmed lumbar disk herniation patients: a comparative effectiveness prospective observational study of 2 age- and sex-matched cohorts treated with either high-velocity, low-amplitude spinal manipulative therapy or imaging-guided lumbar nerve root injections. J Manip Physiol Ther.

[41] Ehrler M, Peterson C, Leemann S, Schmid C, Anklin B, Humphreys BK (2016). Symptomatic, MRI confirmed, lumbar disc Herniations: a comparison of outcomes depending on the type and anatomical axial location of the hernia in patients treated with high-velocity, low-amplitude spinal manipulation. J Manip Physiol Ther.

[42] Wright JG, Swiontkowski MF, Heckman JD (2003). Introducing levels of evidence to the journal. J Bone Joint Surg Am.

[43] Kreiner DS, Hwang SW, Easa JE, Resnick DK, Baisden JL, Bess S, Cho CH, MJ DP, Dougherty P, Fernand R, Ghiselli G, Hanna AS, Lamer T, Lisi AJ, Mazanec DJ, Meagher RJ, Nucci RC, Patel RD, Sembrano JN, Sharma AK, Summers JT, Taleghani CK, Tontz WL, Toton JF (2014). An evidence-based clinical guideline for the diagnosis and treatment of lumbar disc herniation with radiculopathy. Spine J.

[44] Peterson CK, Pfirrmann CW, Hodler J, Leemann S, Schmid C, Anklin B, Humphreys BK (2016). Symptomatic, magnetic resonance imaging-confirmed cervical disk Herniation patients: a comparative-effectiveness prospective observational study of 2 age- and sex-matched cohorts treated with either imaging-guided indirect cervical nerve root injections or spinal manipulative therapy. J Manip Physiol Ther.

[45] Bremner RA (1958). Manipulation in the management of chronic low backache due to lumbosacral strain. Lancet.

[46] Bremner RA, Simpson M (1959). Management of chronic Iumbosacral strain. Lancet.

[47] Krumhansl BR, Nowacek CJ, Grieve GP (1986). Manipulation under anesthesia. Modern manual therapy of the vertebral column.

[48] Barth RJ. Claimant-reported history is not a credible basis or clinical for administrative decision making. AMA Guides Newsletter September/October. 2009:1–7.

[49] Barth RJ. Chronic pain: fundamental scientific considerations, specifically for legal claims. AMA Guides Newsletter. January/February. 2013:1–18.

[50] Carragee EJ (2008). Validity of self-reported history in patients with acute back or neck pain after motor vehicle accidents. Spine J.

[51] Kumar N, Wijerathne SI, Lim WW, Barry TW, Nath C, Liang S. Resistive straight leg raise test, resistive forward bend test and heel compression test: novel techniques in identifying secondary gain motives in low back pain cases. Eur Spine J 2012;21(11):2280-2286. 10.1007/s00586-012-2318-8 PMC3481110 22543413

[52] Weiner SS, Weiser SR, Carragee EJ, Nordin M (2011). Managing nonspecific low back pain: do nonclinical patient characteristics matter?. Spine.

[53] Kongsted A, Kent P, Axen I, Downie AS, Dunn KM. What have we learned from ten years of trajectory research in low back pain? BMC Musculoskelet Disord 2016;17:220. 10.1186/s12891-016-1071-2 PMC4875630 27209166

[54] Globe G, Farabaugh RJ, Hawk C, Morris CE, Baker G, Whalen WM, Walters S, Kaeser M, Dehen M, Augat T (2016). Clinical practice guideline: chiropractic Care for low Back Pain. J Manip Physiol Ther.

[55] Krismer M, van Tulder M (2007). Low back pain Group of the Bone and Joint Health Strategies for Europe project. Strategies for prevention and management of musculoskeletal conditions. Low back pain (non-specific). Best Pract Res Clin Rheumatol.

[56] Bigos SJ, Battié MC, Spengler DM, Fisher LD, Fordyce WE, Nachemson AL HT, Wortley MD (1991). A prospective study of work perceptions and psychosocial factors affecting the report of back injury. Spine.

[57] Hicks GE, Fritz JM, Delitto A, SM MG (2005). Preliminary development of a clinical prediction rule for determining which patients with low back pain will respond to a stabilization exercise program. Arch Phys Med Rehabil.

[58] Mehling WE, Ebell MH, Avins AL, Hecht FM (2015). Clinical decision rule for primary care patient with acute low back pain at risk of developing chronic pain. Spine J.

[59] Flynn T, Fritz J, Whitman J, Wainner R, Magel J, Rendeiro D, Butler B, Garber M, Allison S (2002). A clinical prediction rule for classifying patients with low back pain who demonstrate short-term improvement with spinal manipulation. Spine.

[60] Don AS, Carragee EJ (2009). Is the self-reported history accurate in patients with persistent axial pain after a motor vehicle accident?. Spine J.

[61] Barth RJ. Determining injury-relatedness, work-relatedness, and claim-relatedness. AMA Guides Newsletter. 2012:1–10.

[62] Feinberg SD, Brigham CR, Ensalada L. Assessing impairment and disability in the pain patient. AMA Guides Newsletter January/February. 2016:3–10.

[63] Bigos S, Bowyer O, Braen G (1994). *Acute Low Back Problems in Adults. Clinical Practice Guideline No. 14.* AHCP0R publication no. 95–0642.

[64] Gorrell LM, Engel RM, Brown B, Lystad RP (2016). The reporting of adverse events following spinal manipulation in randomized clinical trials-a systematic review. Spine J.

[65] Swait G, Finch R (2017). What are the risks of manual treatment of the spine? A scoping review for clinicians. Chiropr Man Therap.

[66] Cassidy JD, Boyle E, Côté P, He Y, Hogg-Johnson S, Silver FL, Bondy SJ (2009). Risk of vertebrobasilar stroke and chiropractic care: results of a population-based case-control and case-crossover study. J Manip Physiol Ther.

[67] Cassidy JD, Boyle E, Côté P, Hogg-Johnson S, Bondy SJ, Haldeman S (2017). Risk of carotid stroke after chiropractic care: a population-based case-crossover study. J Stroke Cerebrovasc Dis.

[68] Testai FD, Gorelick PB. An unusual cause of vertebral artery dissection: esophagogastroduodenoscopy. Stroke Res Treat. 2010;2010. 10.4061/2010/915484 PMC2934772 20847949

[69] Wuest S, Symons B, Leonard T, Herzog W (2010). Preliminary report: biomechanics of vertebral artery segments C1-C6 during cervical spinal manipulation. J Manip Physiol Ther.

[70] Herzog W, Tang C, Leonard T (2015). Internal carotid artery strains during high-speed, low-amplitude spinal manipulations of the neck. J Manip Physiol Ther.

[71] Hebert JJ, Stomski NJ, French SD, Rubinstein SM (2015). Serious adverse events and spinal manipulative therapy of the low back region: a systematic review of cases. J Manip Physiol Ther.

[72] Shekelle PG, Adams AH, Chassin MR, Hurwitz EL, Brook RH (1992). Spinal manipulation for low-back pain. Ann Intern Med.

[73] Owens WD, Felts JA, Spitznagel EL (1978). ASA physical status classifications: a study of consistency of ratings. Anesthesiology.

[74] Qaseem A, Snow V, Fitterman N, Hornbake ER, Lawrence VA, Smetana GW, Weiss K, Owens DK, Aronson M, Barry P, Casey DE, Cross JT, Fitterman N, Sherif KD, Weiss KB (2006). Clinical efficacy assessment Subcommittee of the American College of physicians. Risk assessment for and strategies to reduce perioperative pulmonary complications for patients undergoing noncardiothoracic surgery: a guideline from the American College of Physicians. Ann Intern Med.

[75] Hurwitz EE, Simon M, Vinta SR, Zehm CF, Shabot SM, Minhajuddin A, Abouleish AE (2017). Adding examples to the ASA-physical status classification improves correct assignment to patients. Anesthesiology.

[76] American Academy of osteopathy consensus statement for osteopathic manipulation of somatic dysfunction under anesthesia and conscious sedation. The American Academy of Osteopathy Journal. 2005;15(2):26–7.

[77] Association of Chiropractic Colleges. Informed consent guideline. http://www.chirocolleges.org/resources/informed-consent-guideline/. Accessed 17 Nov 2017.

[78] Wilson JN, Ilfeld FW (1952). Manipulation of the herniated intervertebral disc. Am J Surg.

[79] Siehl D, Bradford WG (1952). Manipulation of the low back under general anesthesia. J Am Osteopath Assoc..

[80] Ewer EG (1953). Manipulation of the spine. J Bone Joint Surg Am.

[81] Siehl D. Manipulation of the spine under general anesthesia. J Am Osteopath Assoc. 1963;62:881–7. 13988981

[82] Tospon HN (1972). Manipulation of the lumbar spine under anesthesia. The Orthop.

[83] Morey LW (1973). Osteopathic manipulation under general anesthesia. J Am Osteopath Assoc..

[84] Scherrer H (1977). Hernia of intervertebral disc: treatment by manipulation under general anesthesia. Z Orthop Ihre Grenzgeb.

[85] Spine: Levels of evidence for primary research question. https://www.spine.org/Documents/ResearchClinicalCare/LevelsOfEvidence.pdf. Accessed 17 Nov 2017.

[86] American Heart Association. Understanding blood pressure readings. http://www.heart.org/HEARTORG/Conditions/HighBloodPressure/KnowYourNumbers/Understanding-Blood-Pressure-Readings_UCM_301764_Article.jsp#.Wg7QR7pFyP8. Accessed 17 Nov 2017.

